# Thin-slice computed tomography enables to classify pulmonary subsolid nodules into pre-invasive lesion/minimally invasive adenocarcinoma and invasive adenocarcinoma: a retrospective study

**DOI:** 10.1038/s41598-023-33803-x

**Published:** 2023-04-28

**Authors:** Min Li, Lei Zhu, Yilv Lv, Leilei Shen, Yuchen Han, Bo Ye

**Affiliations:** 1 Department of Thoracic Surgery, Shanghai Chest Hospital, Shanghai Jiaotong University, 241 Huaihai West Road, Xuhui District, Shanghai, 200030 China; 2 Department of Radiology, Shanghai Municipal Hospital of Traditional Chinese Medicine, Shanghai University of Traditional Chinese Medicine, Shanghai, China; 3 Department of Pathology, Shanghai Chest Hospital, Shanghai Jiaotong University, 241 Huaihai West Road, Xuhui District, Shanghai, 200030 China; 4 Department of Radiology, Shanghai Chest Hospital, Shanghai Jiaotong University, Shanghai, China

**Keywords:** Cancer imaging, Lung cancer

## Abstract

The aim was to investigate the ability of thin-slice computed tomography (TSCT) to differentiate invasive pulmonary adenocarcinomas (IACs) from pre-invasive/minimally invasive adenocarcinoma (AAH-MIAs), manifesting as subsolid nodules (SSNs) of diameter less than 30 mm. The CT findings of 810 patients with single subsolid nodules diagnosed by pathology of resection specimens were analyzed (atypical adenomatous hyperplasia, n = 13; adenocarcinoma in situ, n = 175; minimally invasive adenocarcinoma, n = 285; and invasive adenocarcinoma, n = 337). According to the classification of lung adenocarcinoma published by WHO classification of thoracic tumors in 2015, TSCT features of 368 pure ground-glass nodules (pGGN) and 442 part-solid nodules (PSNs) were compared AAH-MIAs with IACs. Logistic regression and receiver operating characteristic (ROC) curve analyses were performed. In pGGNs, multivariate analysis of factors found to be significant by univariate analysis revealed that higher mean-CT values (*p* = 0.006, OR 1.006, 95% CI 1.002–1.010), larger tumor size (*p* < 0.001, OR 1.483, 95% CI 1.304–1.688) with air bronchogram and non-smooth margins were significantly associated with IACs. The optimal cut-off tumor diameter for AAH-MIAs lesions was less than 10.75 mm (sensitivity, 82.8%; specificity, 80.6%) and optimal cut-off mean-CT value − 629HU (sensitivity, 78.1%; specificity, 50.7%). In PSNs, multivariate analysis of factors found to be significant by univariate analysis revealed that smaller tumor diameter (*p* < 0.001, OR 0.647, 95% CI 0.481–0.871), smaller size of solid component (*p* = 0.001, OR 83.175, 95% CI 16.748–413.079),and lower mean-CT value of solid component *(p* < 0.001, OR 1.009, 95% CI 1.004–1.014) were significantly associated with AAH-MIAs (*p* < 0.05). The optimal cut-off tumor diameter, size of solid component, and mean-CT value of solid component for AAH-MIAs lesions were less than 14.595 mm (sensitivity, 71.1%; specificity, 83.4%), 4.995 mm (sensitivity, 97.8%; specificity, 92.3%) and − 227HU (sensitivity, 65.6%; specificity, 76.3%), respectively. In subsolid nodules, whether pGGN or PSNs, the characteristics of TSCT can help in distinguishing IACs from AAH-MIAs.

## Introduction

Lung cancer is the leading cause of cancer death among men and the second-leading cause among women worldwide^1^. Adenocarcinoma is the most prevalent histological type of lung cancer. In recent years, with developments in CT imaging and applications, the rate of detection of lung subsolid nodules (SSNs) has greatly increased. On thin-slice computed tomography (TSCT), GGNs appear as a hazy nodular increased attenuation of lung with preservation of bronchial and vascular margins^2^. Pulmonary nodules are classified into three categories: solid, pure ground-glass, and part-solid nodules (PSNs) based on thin-section CT. SSNs include pure ground-glass nodules (pGGNs) and PSNs. The 4th edition of the World Health Organization (WHO) Book on Classification of Thoracic Tumors has produced criteria for subdividing lung adenocarcinomas into pre-invasive lesion, including atypical adenomatous hyperplasia (AAH) and adenocarcinoma in situ (AIS), minimally invasive adenocarcinoma (MIA), and invasive adenocarcinoma (IAC)^3^. IAC has been further divided into lepidic predominant adenocarcinoma (LPA), acinar predominant adenocarcinoma (APA), papillary predominant adenocarcinoma (PPA), micropapillary predominant adenocarcinoma (MPA), solid predominant adenocarcinoma (SPA), and other less frequently occurring variants of invasive adenocarcinoma. AAH/AIS and MIA are much less likely than IAC to spread to regional lymph nodes or distant organs and have an almost 100% 5-year recurrence-free survival^4^. Infiltration by lung adenocarcinoma affects its stage and accordingly the prognosis. Compared with AIS and MIA, IAC is more aggressive and has a worse prognosis^5^. Lobectomy is usually performed for IAC, while segmental or wedge resection is performed for AAH-MIA. patients with AIS or MIA usually have less tissue damage and a better prognosis. It is therefore very important to differentiate between AIS-MIA and IAC before surgery in clinical practice. In lung adenocarcinoma, CT imaging features are closely related to the pathology. Various TSCT features can help differentiate SSNs including tumor size, mean-CT value, mean-CT value of solid component, size of solid component, bubble-like sign, bronchial sign and others. For patients with lung adenocarcinoma, early diagnosis is also essential for the surgical process and prognosis.

The purpose of this study was to comprehensively analyze the correlations between imaging features and pathological classification of pulmonary adenocarcinoma presenting as SSNs. We believe that preoperative TSCT imaging analysis is helpful in distinguishing IAC from pre-invasive/MIA and enabling better formulation of appropriate treatment options.

## Materials and methods

### Ethical approval

This retrospective was approved by the Institutional Review Board for clinical research of Shanghai Chest Hospital and carried out according to the principles embodied in the Declaration of Helsinki. The need for informed patient consent was waived by the Institutional Review Board in view of the anonymity of the data and the retrospective observational nature of this study. All methods were carried out in accordance with the relevant guidelines and regulations.

### Patients

**Figure 1:**
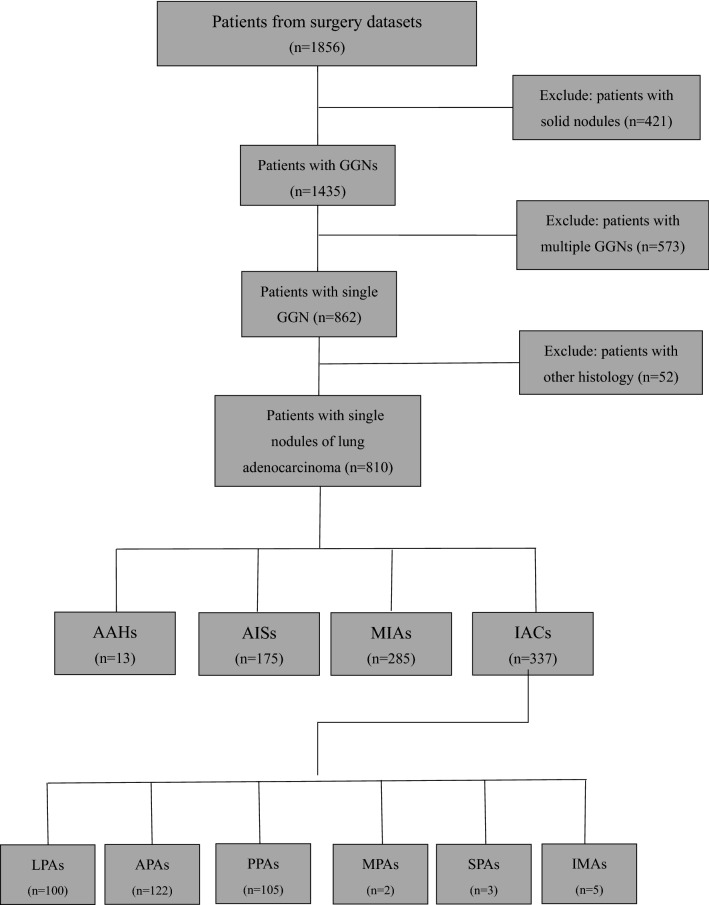
Flow chart showing the process for selection for the study. *GGN* ground-glass nodules, *AAH* atypical adenomatous hyperplasia, *AIS* adenocarcinoma in situ, *MIA* minimally invasive adenocarcinoma, *IAC* invasive pulmonary adenocarcinoma, *LPA* lepidic predominant invasive adenocarcinoma, *APA* acinar predominant invasive adenocarcinoma, *PPA* papillary predominant invasive adenocarcinoma, *MPA* micropapillary predominant adenocarcinoma, *SPA* solid predominant adenocarcinoma, *IMA* invasive mucinous adenocarcinoma.

Electronic medical records data, including radiological findings, of 1856 consecutive patients who underwent curative resection of lung adenocarcinoma in Shanghai Chest Hospital Affiliated to Shanghai Jiaotong University from January 2014 to December 2017 were reviewed. Our inclusion criteria were as follows: (1) availability of chest CT scan with thin-slice thickness within one week prior to surgery; (2) CT detection of pGGNs (having only a ground glass opacity [GGO] component or PSNs (having both GGO and consolidation components and solid nodules having only a consolidation component)^6^; (3) SSNs longest diameter ≤ 30 mm on axial images; and (4)completed clinical and imaging data and pathological correlation. The exclusion criteria were: (1) CT detection of solid nodules; (2) presence of multiple nodules; (3) other histological diagnosis except lung adenocarcinoma, such as benign nodule, squamous-cell carcinoma, and carcinoid. Finally, 810 patients with SSNs were included. The process for selecting eligible patients is shown in Fig. 1.

The lesions of the 810 eligible patients were diagnosed by surgery and pathological examination as 13 AAH, 175 AIS, 285 MIA and 337 IACs. The patients’ ages ranged from 15 to 84 years, with an average of (55.09 ± 11.47) years. There were 282 male and 528 female patients. The 337 IACs comprised 100 LPA, 122 APA, 105 PPA, two MPA, three SPA, and five IMA. There were no cases of colloid adenocarcinoma, fetal adenocarcinoma, or enteric adenocarcinoma.

### CT examinations

All CT examinations were performed using a Philips Brilliance 64-slice helical CT (Philips Brilliance, Cleveland, OH, USA) preoperatively. Scanning was performed from the lung apex to the base. The scanning parameters were as follows: pitch 1.08, width of collimation 64 mm × 0.625 mm, slice thickness 0.625–1 mm, tube voltage 120 kV, current 200 mA, 512 × 512 image matrix, FOV 35 cm, and speed 0.6 s/r. The scan of the target was performed as for conventional CT scanning, the pitch being 1.08, width of collimation 64 mm × 0.625 mm, most slice thicknesses 0.625 mm with a few of 1 mm, tube voltage 120 kV, current 200 mA, FOV 20 cm, and speed 0.6 s/r. All CT images were captured at the center of the lung window: − 700 HU and window width, 1200 HU; and mediastinal window center: 60 HU and window width 450 HU. All examinations were performed unenhanced.

### Analysis of CT findings

Two double-blinded radiologists read the images independently and selected as close to the same size of the region of interest as possible for measuring the CT value of the lesion on a multi-plane reconstruction. Radiologists used circular ROI. When drawing ROIs for pGGNs, radiologists used 60% area or diameter to include the whole nodule, avoiding partial volume effect at the edges as much as possible. For PSNs, measurement of CT values of the whole nodule and the solid component is similar to that of pGGNs, but they were evaluated independently. Measurements were performed without calcification, necrosis, or cavities. The averages of the findings of the two radiologists were calculated. Where there were large discrepancies, the radiologists examined the images together until they reached agreement.

### Evaluation of TSCT features

TSCT-identified features of each nodule were classified as follows: (1) pattern (pGGN or PSNs); (2) location: right upper lobe (RUL), right middle lobe (RML), right lower lobe (RLL), left upper lobe (LUL), or left lower lobe (LLL); (3) air bronchogram; (4) bubble-like sign; (6) tumor margin (any lobulated, spiculated, or both). Quantitative variables include: (1) diameters (largest dimension visible on transverse CT sections^7^; mm) of the solid component and whole tumor; (2) mean CT value (HU) of the solid component and whole tumor; (3) ratio of solid component to the whole tumor (C/T ratio), all of these being measured at the maximum diameter of the solid component and whole nodule. All collected GGNs were subdivided into pGGNs and PSNs for further imaging analysis. The measurements were repeated twice by two radiologists and the mean values recorded. Any differences were settled by consensus.

### Pathological evaluation

The surgically resected specimens were fixed in 10% formalin, embedded in paraffin, sectioned with a microtome, and stained with hematoxylin and eosin(H&E). All tissue sections were analyzed by two pathologists, and a consensus reached. In accordance with 2015 WHO classification of thoracic tumors, pre-invasive lesions (AAHs and AISs) were defined as lesions showing no stromal, vascular, or pleural invasion whereas IAs were defined as adenocarcinomas containing an invasive component, that is, minimally invasive adenocarcinoma and invasive adenocarcinoma of various subtypes, including lepidic predominant, acinar predominant, and papillary predominant adenocarcinomas.

### Statistical analysis

Data were analyzed using SPSS22.0 statistical software (Armonk, NY, USA). Pearson’s χ^2^ test was used to compare qualitative variables and *t*-tests and Z-tests to compare quantitative variables. The variables that exhibited statistically significant differences were included in multivariate logistic regression analysis. Additionally, the receiver operating characteristics (ROC) of variables with statistically significant differences in multivariate logistic regression were analyzed. The area under the curve (AUC), sensitivity, and specificity under the curve were obtained for different cut-off points, after which the value corresponding to the top-left point in the maximum area under the curve was selected as the optimal cut-off point. p < 0.05 was considered to denote statistical significance.

ROC curves were analyzed using a commercial software package (MEDCALC 11.3.8; MEDCALC software, Ostend, Belgium).

## Results

### Morphologic features and univariate analysis on all patients

Comparisons of the features of AAH-MIAs (n = 473) and IACs (n = 337) on TSCT are shown in Table 1. Univariate analysis showed statistically significant differences in age (p < 0.001), sex (p = 0.002), size of solid component (p < 0.001), tumor size (p < 0.001), C/T ratio (p < 0.001), bubble-like sign (p < 0.001), air bronchogram (P = 0.009), margin (p < 0.001), and surgical procedure (p < 0.001) between AIS-MIAs and IACs. The only assessed variable that was not significant was location (p = 0.176).

**Table 1:** Results of univariate analysis of indicated variables in patients with SSNs**.**

	Pre-invasive/minimally invasive (n = 473) (% or range)	Invasive (n = 337) (% or range)	p
Age	51.92 ± 11.79	59.55 ± 9.34	< 0.001
Gender			0.002
Male	144 (30.4)	138 (40.9)	
Female	329 (69.6)	199 (59.1)	
Location			0.176
LLL	67 (14.2)	30 (8.9)	
LUL	132 (27.9)	97 (28.8)	
RLL	57 (12.1)	52 (15.4)	
RML	35 (7.4)	26 (7.7)	
RUL	182 (38.5)	132 (39.2)	
Tumor size (mm)	9.00 (7.16–11.05)	17.18 (13.00–22.22)	< 0.001
Mean CT value (HU)	− 585.94 (− 666.50 to − 491.92)	− 421.79 (− 532.50 to − 285.75)	< 0.001
Size of solid component (mm)	3.40 (2.56–4.40)	8.30 (6.42–11.51)	< 0.001
Mean CT value of solid component (HU)	− 328.73 (− 413.50 to − 229.43)	− 158.00 (− 261.03 to − 78.67)	< 0.001
C/T ratio	0.34 (0.26–0.41)	0.50 (0.40–0.65)	< 0.001
Pattern			< 0.001
pGGN	304 (64.3)	64 (19.0)	
PSN	169 (35.7)	273 (81.0)	
Bubble-like sign	70 (14.8)	74 (22.0)	0.009
Air bronchogram	56 (11.8)	91 (27.0)	< 0.001
Margin			< 0.001
Smooth	340 (71.9)	50 (14.8)	
Lobular	71 (15.0)	99 (29.4)	
Spiculated	43 (9.1)	84 (24.9)	
Lobular + spiculated	19 (4.0)	104 (30.9)	
Operation type			< 0.001
Segmentectomy	196 (41.4)	32 (9.5)	
Partial resection	105 (22.2)	64 (19.0)	
Lobectomy	172 (36.4)	241 (71.5)	

*C/T ratio* ratio of solid component to the whole tumor.

There were 473 AAH-MIAs and 337 IACs. CT identified 442 tumors as PSNs, including no AAHs (0%), 43 AISs (24.6%), 126 MIAs (44.2%), 69 LPAs (69%), 110 APAs (90.2%), 84 PPAs (80%), two MPAs (100%), three SPAs (100%), and five IMAs (100%), as shown in Table 2. To fine-tune our assessment, we compared quantitative variables of AAH-MIAs and IACs in pGGNs and PSNs.

**Table 2:** Distribution of different pathological subtypes in pGGNs and PSNs.

	AAH	AIS	MIA	LPA	APA	PPA	MPA	SPA	IMA
pGGN	13	132	159	31	12	21	0	0	0
PSN	0	43	126	69	110	84	2	3	5
Total	13	175	285	100	122	105	2	3	5

*AAH* atypical adenomatous hyperplasia, *AIS* adenocarcinoma in situ, *APIA* acinar predominant invasive adenocarcinoma, *GGN* ground-glass nodules, *LPIA* lepidic predominant invasive adenocarcinoma, *MIA* minimally invasive adenocarcinoma, *PPIA* papillary predominant invasive adenocarcinoma.

### Analyses regarding pure GGNs

**Figure 2:**
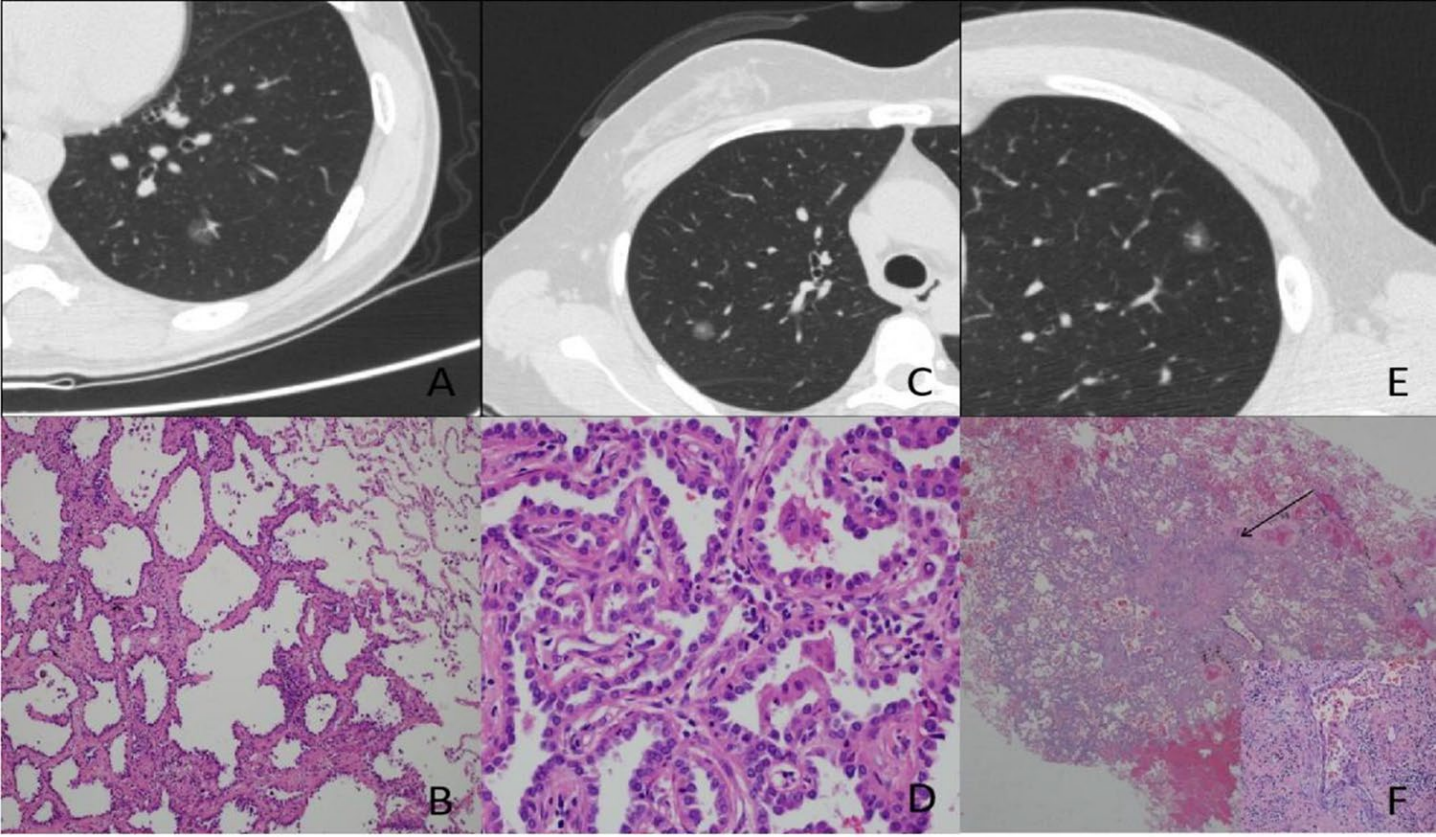
A 48-year-old woman with an AIS in the left lower lobe of her lung. Axial target scanning image showing a 9.8-mm diameter pGGN with CT value of − 662 HU (**A**). Photomicrograph of section from the resected specimen showing tumor cells growing along thickened alveolar walls (lepidic growth) (**B**) (hematoxylin and eosin [H & E], × 100). A 47-year-old woman with an AIS in the right upper lobe of her lung. Axial target scanning image showing a 8.4-mm diameter PSN with a solid component of diameter 1.7-mm and CT value − 380 HU (**C**). Photomicrograph of section from the resected specimen showing alveolar collapse, alveolar compression, and formation of pseudo-infiltration (**D**) (H & E, × 400). A 28-year-old man with an MIA in the left upper lobe of his lung. Axial target scanning image showing an 11.4-mm diameter PSN with a solid component of diameter 4.5-mm and CT value − 97 HU (**E**). Photomicrograph of section from the resected specimen showing a lepidic predominant lesion consisting of predominantly lepidic tumor growth with foci of invasive acinar components of less than 5 mm (arrow) (**F**) (H & E, × 20; high magnification [× 200] inset).

Results of univariate and multivariate logistic regression analysis of pGGNs are shown in Table 3. It was found that larger tumor size (p < 0.001), higher mean CT value (p = 0.006), more air bronchogram (p < 0.001), and more irregular tumor margins (lobular [p < 0.001], spiculated [p < 0.001], and lobular + spiculated (p < 0.001)] were significantly more strongly associated with IACs than with AAH-MIAs (Fig. 2).

**Table 3:** Results of univariate and multivariate analysis of indicated variables in pGGNs.

Variables	Univariate(Z/t/χ^2^))	*p*	Multivariate OR (95% CI)	*p*
Age	− 5.822	0.000		
Gender	8.168	0.004		
Male			1.000	
Female			0.377 (0.152–0.939))	0.036
Tumor size (mm)	− 9.011	0.000	1.483 (1.304–1.688))	< 0.001
Mean CT value (HU)	− 4.376	0.000	1.006 (1.002–1.010))	0.006
Bubble-like sign	10.357	0.001		
Air bronchogram	32.963	0.000	7.878 (2.923–21.232)	< 0.001
Margin	103.565	0.000		
Smooth			1.000	
Lobular			18.512 (5.699–60.133)	< 0.001
Spiculated			16.895 (4.538–62.897)	< 0.001
Lobular + spiculated			53.960 (12.193–238.793)	< 0.001
Operation type	9.561	0.008		
Segmentectomy				
Partial resection				
Lobectomy				

*C/T ratio* ratio of solid component to the whole tumor.

Table 4 shows results of ROC analysis of tumor size and mean CT values for AAH-MIA and IAC in pGGNs. The cut-off, AUC, sensitivity, specificity of tumor size were 10.75 mm; 0.858; 82.8%; and 80.6%, respectively, whereas the cut-off, AUC, sensitivity, specificity of mean CT were − 629 HU; 0.674; 78.1%; and 50.7%, respectively. The AUC value is 0.881 when tumor size and mean CT value are combined.

**Table 4:** Findings derived from ROC curve of pure ground-glass nodules.

	AUC	95% CI	Cutoff value	Sensitivity	Specificity
Tumor size (mm)	0.858	0.802–0.914	10.75	82.8	80.6
Mean CT value (HU)	0.674	0.602–0.746	− 629.315	78.1	50.7
Tumor size + mean CT value	0.881	0.828–0.934		78.1	87.5

### Analyses regarding PSNs

**Figure 3:**
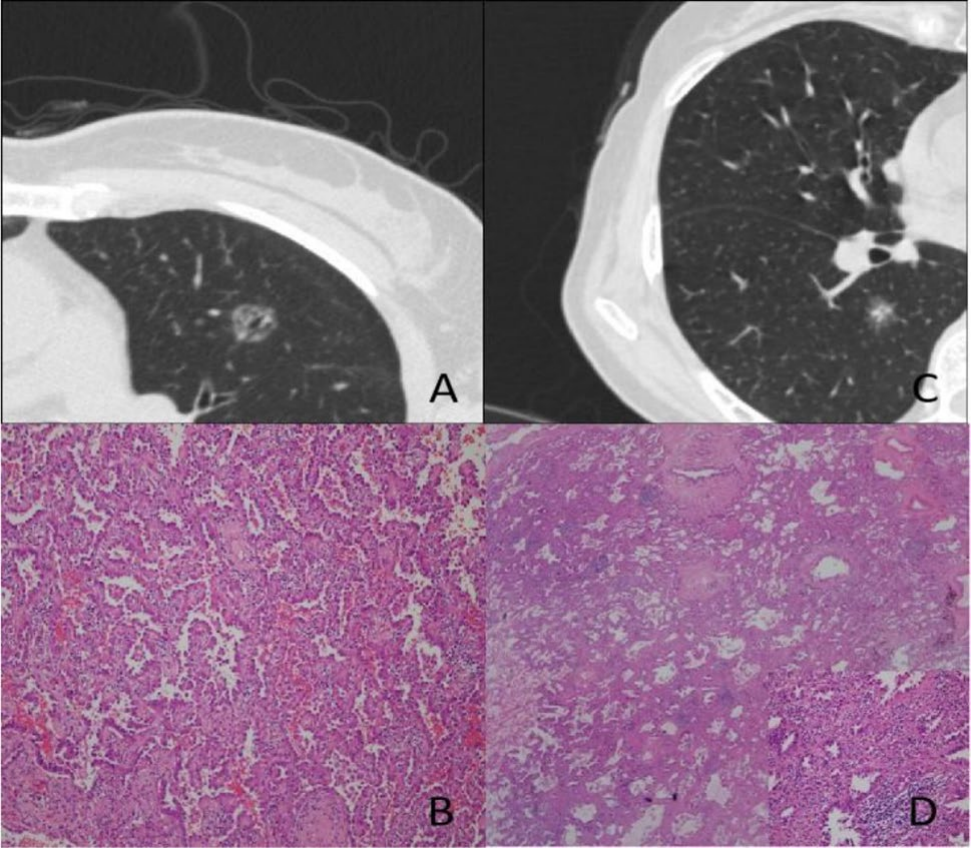
A 50-year-old woman with a PPA in the left upper lobe of her lung. Axial target scanning image showing a 16.4-mm diameter pGGN with air bronchogram and a CT value of − 643 HU (**A**). Photomicrograph of section from the resected specimen showing papillary adenocarcinoma cells growing along central fibrovascular cores (**B**) (H & E, × 100). A 56-year-old woman with an APA in the right lower lobe of her lung. Axial target scanning image showing a 15.9-mm diameter PSN with a solid component of diameter 6.7-mm, CT value of − 176 HU, and lobulation and spiculation (**C**). Photomicrograph of section of the resected specimen showing some of the tumor cells growing along the surface of the alveolar wall and invasive adenocarcinoma component of acinar type measuring larger than 5 mm (arrow) (**D**) (H & E), × 20; high magnification [× 200) inset).

Results of univariate and multivariate logistic regression analysis of PSNs are shown in Table 5. It was found that larger tumor size (p < 0.001), size of solid component (p = 0.001), higher mean CT value of solid component (p < 0.001), and more irregular tumor margin lobular [p < 0.001], spiculated [p = 0.005], lobular + spiculated [p = 0.001]) were significantly more strongly associated with IACs than with AAH-MIAs (Fig. 3).

**Table 5:** Results of univariate and multivariate analysis of indicated factors in PSNs.

Variables	Univariate(Z/t/χ^2^)	*p*	Multivariate OR (95% CI)	*p*
Age	− 6.935	0.000	1.056 (1.001–1.115)	0.047
Gender	2.773	0.096		
Male				
Female				
Tumor size (mm)	− 12.524	0.000	0.647 (0.481–0.871)	0.004
Mean CT value (HU)	− 7.780	0.000		
Size of solid component (mm)	− 17.242	0.000	83.175 (16.748–413.079)	< 0.001
Mean CT Value of solid component (HU)	− 9.450	0.000	1.009 (1.004–1.014)	0.001
C/T ratio	− 11.950	0.000		
Bubble-like sign	1.361	0.243		
Air bronchogram	3.659	0.056		
Margin	121.989	0.000		
Smooth			1.000	
Lobular			38.796 (5.373–280.098)	< 0.001
Spiculated			15.826 (2.315–108.190)	0.005
Lobular + spiculated			23.719 (3.535–159.156)	0.001
Operation type	75.864	0.000		
Segmentectomy				
Partial resection				
Lobectomy

Table 6 shows results of ROC analysis of tumor size, mean CT value of the solid component, and size of the solid component for AAH-MIA and IAC in PSNs. The cut-off, AUC, sensitivity, specificity of tumor size were 14.595 mm; 0.854; 71.1%; 83.4%, respectively.The cut-off, AUC, sensitivity, specificity of mean CT value of the solid component were − 227HU; 0.767; 65.6%; and 76.3%, respectively, whereas the cut-off, AUC, sensitivity, specificity of size of the solid component were 4.995 mm; 0.988; 97.8%; and 92.3%, respectively. The AUC value is 0.992 when the tumor size, size of solid component and mean CT value of solid component are combined.

**Table 6:** Findings derived from ROC curve of part-solid ground-glass nodules.

	AUC	95% CI	Cutoff value	Sensitivity	Specificity
Tumor size (mm)	0.854	0.819–0.889	14.595	71.1	83.4
Size of solid component (mm)	0.988	0.981–0.995	4.995	97.8	92.3
Mean CT value of solid component (HU)	0.767	0.723–0.811	− 227.145	65.6	76.3
Tumor size + size of solid component + mean CT value of solid component	0.992	0.987–0.997		93.0	97.6

## Discussion

The incidence and mortality of lung cancer are the highest in both the world and China. About half of patients with lung cancer die within 1 year of diagnosis^1^. One important reason for this high mortality rate is that lung cancer is characteristically not diagnosed early, thus missing the best time for treatment: if lung adenocarcinomas are correctly diagnosed and treated early, the 5 year survival rate can be increased to 50%^8^. In the new classification of lung adenocarcinoma introduced in 2011, the disease-related survival rate of patients with MIA lesions is close or equal to 100% provided the patient undergoes complete surgical resection. It is therefore extremely important to accurately distinguish between pre-invasive lesions/MIA and invasive lung adenocarcinoma preoperatively. A large proportion of lung adenocarcinomas present as SSNs on CT scans; therefore, it is extremely important significance to recognize the CT features of SSNs when selecting surgical procedures.

Kim et al.^9^ have found that pGGNs are not necessarily AIS and vice versa. There is reportedly considerable overlap in the size and visual morphology of IPAs and pre-invasive lesions^10^. Therefore, we subdivided all SSNs into pGGNs and PSNs, then compared the imaging features of each subgroup’s pathological subtypes. A previous study^11^ has shown that the size of SSNs is strongly associated with malignant change. Even pGGNs are more likely than not to be IAC when larger than 16.4 mm^12^. Cho et al.^13^ found that larger diameter is an independent risk factor for malignancy. Tumor size is used in staging of lung cancer and has been shown to be a predictor of prognosis^14^. In our study, we also found by both univariate and multivariate analysis that, for both pGGNs and PSNs, tumor diameter contributed significantly to differentiate pre-invasive lesions from IAC. A previous study^15^ has shown that a cut-off value of 10.5 mm is optimal for distinguishing pre-invasive lesions from invasive lesions with pGGNs, with a sensitivity of 86.3% and a specificity of 61.9%, and IPAs have relatively low specificity for all SSNs if the lesion size cut-off is 12 mm. In this study, we used a relatively large number of samples (810 patients) and compared the CT features of lung adenocarcinoma manifesting as pGGNs and PSNs. A 10.75 mm cut-off value was optimal for pGGNs, reaching 82.8% sensitivity and 80.6% specificity. Meanwhile, the 14.595 mm cut-off value was optimal for PSNs, reaching 71.1% sensitivity and 83.4% specificity.

However, other imaging features besides size must be assessed when predicting the pathological diagnosis of an SSN. Especially with PSNs, the size of the solid component is a better predictor of prognosis than the overall tumor size in patients with lung cancer^16–18^. In our study, we found that the bigger the solid component, the greater the likelihood of malignancy and invasiveness; this is consistent with the findings of previous studies. The CT optimal cut-off value was 4.995 mm, which yielded a sensitivity of 76.9% and a specificity of 94.70%. Thus, the size of the solid component is the most important determinant of subsequent treatment.

Because pGGNs have no solid component, the naked eye cannot distinguish different CT values within them; therefore, the mean CT value of the ground-glass component of pGGNs is usually evaluated. In AAH and AIS, few alveolar structures are destroyed, and the tumor cells are not densely arranged. Thus, the air content in the tumor tissue is relatively constant. In MIA, the concentration of tumor cells and tumor tissue as a whole is greater, there is therefore greater accumulation of exfoliated cells in the alveolar cavities of the tumor, and the local air content is relatively low. In IAC, there is significantly less air in the affected area; thus, the density of IAC is significantly higher than that of AAH, AIS and MIA. In our study, there was a statistically significant difference in density between AAH-MIA and IAC, a ROC curve showing that an optimal CT cut-off value of − 629HU, resulting in a sensitivity of 78.1% and a specificity of 50.7%. A previous CT study has shown that the degree of malignancy and extent of infiltration are in direct proportion to the solid density^19^.We also found that the mean CT value of the whole tumor was an independent predictor of IAC by univariate analysis, however, it was not a statistically significant factor according to multivariate analysis. In contrast, the CT value of solid components was a statistically significance factor according to both univariate and multivariate analysis. ROC curve showed that the optimal CT cut-off value for the solid component was − 227HU, which yielded a sensitivity of 65.6% and a specificity of 76.3%.

We combined multiple factors in the ROC curves of pGGNs and PSNs, respectively, to assess the corresponding AUC results. The findings indicated that combining tumor size and mean CT value resulted in a sensitivity of 78.1% and specificity of 87.5% for pGGNs. Similarly, when the tumor size, size of solid component, and mean CT value of solid component were considered together, the sensitivity for PSNs was 93% and the specificity was 97.6%. Based on these results, combining multiple factors would improve diagnostic accuracy.

According to previous studies^20,21^, air bronchogram are more frequently observed in IACs. The bubble-like sign and air bronchogram in GGNs are imaging features of lung adenocarcinoma^22^. The pathological basis for air bronchograms is that the tumor cells grow along and attach to the alveolar wall, resulting in accumulation of cells in the alveolar cavity, which shows as a ground-glass density in an image. Because the bronchioles are not invaded in the early stages, tumor nodules do not form in their walls; accordingly, their lumen do not get blocked. The pathological basis for the bubble-like sign is mainly that lung structural supports such as alveoli and expanded, and twisted patent bronchioles have not been destroyed or replaced by tumors. In our study, we found significant differences between AAH-MIA and IAC in the frequency of bubble-like signs and air bronchograms. According to binary logistic regression analysis, air bronchograms are an important risk factor for predicting IAC in pGGNs.

Xing et al.^23^ reported that lobulation and spiculation may be associated with pGGOs. Spiculation indicates invasiveness and therefore suggests the transformation to invasive adenocarcinoma^24^; it is the strongest predictor of invasion. In our study, both lobulation and spiculation were identified as independent risk factors for malignant lesions in both pGGNs and PSNs. Because peripheral infiltration of pGGNs is less extensive than that of PSNs, the incidence of lobulation and spiculation in the former is relatively low, even when the lesions are malignant.

Our study had several limitations. First, all patients had undergone surgery; thus, the selection criteria of the thoracic surgons may have caused some selection bias. Second, we did not evaluate the density of the lesions because some of the pGGOs were small, and there were vessels, air, and bronchi within the lesions. Data obtained by manual methods are relatively inaccurate; more precise methods of measuring imaging features of these lesions need to be further studied. Third, we did not evaluate intra-observer variability. Fourth, our results were not externally validated, and the accuracy of validation data will be reported in the future study. Fifth, as less than 20 patients reported smoking status, an important clinicopathological parameter, was not analyzed in this retrospective study. Last, this study only included patients with solitary subsolid nodules; thus, further studies are required in patients with multiple nodules.

## Conclusions

In conclusion, in pGGNs, smaller lesion size, lower mean CT value of lesions, and fewer air bronchogram signs, lobulation and spiculation indicate a stronger likelihood of being AAH-MIAs. In PSNs, AAH-MIAs can be distinguished from IACs by smaller lesion diameter, smaller size of solid component, lower CT value of solid component, and fewer lobulation and spiculation. Although this study had some shortcomings, our results may assist in distinguishing between AAH-MIA and IAC and provide some guidance on management decisions.

## Data Availability

The datasets used and/or analysed during the current study are available from the corresponding author (Bo Ye) on reasonable request.

## Abbreviations

AAH: Atypical adenomatous hyperplasia
AIS: Adenocarcinoma in situ
MIA: Minimally invasive adenocarcinoma
IAC: Invasive adenocarcinoma
pGGN: Pure ground-glass nodule
PSN: Part-solid ground-glass nodule
SSN: Subsolid nodule
TSCT: Thin-slice computed tomography
